# Intervention components in the self-management of Parkinson’s: a mixed-methods synthesis of qualitative and quantitative evidence

**DOI:** 10.1186/s12913-023-10436-4

**Published:** 2024-01-17

**Authors:** Megan Armstrong, Kate Walters, Nathan Davies, Danielle Nimmons, Jennifer Pigott, Joy Read, Anette Schrag

**Affiliations:** 1 Department of Primary Care and Population Health, University College London, Royal Free Campus, Rowland Hill Street, London, NW3 2PF UK; 2 Institute of Neurology, University College London, London, UK

**Keywords:** Parkinson's, Synthesis, Self-management

## Abstract

**Introduction:**

Self-management interventions consist of multiple components to support people in the management of medical, emotional, and behavioural aspects of their condition, and aim to improve quality of life, function, and other outcomes. A systematic review of self-management interventions in Parkinson’s showed no conclusive evidence for effectiveness of specific self-management approaches in Parkinson’s to date but identified several potentially useful components.

**Aim:**

To identify the key required components for self-management in people with Parkinson’s by synthesising evidence from a body of primary qualitative evidence and systematic reviews, and to explore which of these key components should be incorporated into trials of self-management in Parkinson’s.

**Method:**

A mixed-methods synthesis was conducted. We combined data from two primary qualitative studies and a systematic review of qualitative studies that focused on self-management in Parkinson’s to identify key intervention components. These were then mapped onto the results of a systematic review of Randomised Controlled Trials (RCTs) using matrices. First, data were extracted from the qualitative studies with people with Parkinson’s and healthcare professionals on the key self-management components in this population. Second, a matrix table was created to map the identified Parkinson’s specific self-management components against potential effectiveness from published RCTs of self-management interventions.

**Results:**

Synthesis of qualitative data identified 15 potential self-management components. These 15 components included components needed to start self-managing (e.g., information, skill acquirement) and components needed to maintain self-managing (e.g., self-motoring, increasing motivation). From 18 RCTs, interventions varied in how many components were included (range 1–10). Trials reporting significant beneficial effects of their intervention included a higher number of components (4 or more self-management components) than trials without significant findings (1–3 self-management components).

**Conclusion:**

Fifteen key self-management components were identified that should be incorporated into interventions or programs of self-management in Parkinson’s. No current trial has incorporated all aspects, but a higher number of these key components appears to make trials of self-management interventions more likely to be successful.

## Introduction

Parkinson’s is a chronic and progressive condition causing motor disability, reduced mobility, and falls. It is also associated with a complex range of disabling and distressing non-motor symptoms including cognitive impairment, apathy, depression, anxiety, psychosis, bowel and bladder dysfunction, fatigue, and pain [1, 2]. However, many aspects of Parkinson’s can be managed resulting in better symptom control and improved quality of life. Due to the complex and chronic nature of Parkinson’s, this requires an element of self-management whereby the person with the condition takes an active role in managing the medical and emotional aspects and engages with preventative health behaviours [3]. Supporting people and their carers in self-management in a variety of long-term health conditions has been found to be associated with improved health outcomes such as reduced pain, and improved quality of life and functioning [4]. Evidence for self-management interventions for people with Parkinson’s has been mixed with no conclusive evidence of effectiveness of the approaches tested but have shown some promise in improving well-being, function and quality of life [5].

Self-management involves management of medical, emotional, and behavioural aspects of their condition [6] Interventions use multiple components, such as behaviour change techniques, support with medication management, and goal setting, to support people in self-management [7]. A taxonomy of 14 active self-management components has been proposed to inform the development of self-management interventions for long-term conditions [8]. The taxonomy was originally created following synthesis of systematic reviews of trials on self-management and then qualitatively explored in a group of cancer survivors. The taxonomy included components on access to information on the condition and lifestyle advice, resources, and clinical action plans and reviews; equipment and monitoring of the condition; training on everyday activities, self-management, working with HCPs, psychological strategies; and practical and social support. The authors of the taxonomy specified that it is not intended that all 14 components would be used in all interventions as this will be influenced by the condition and outcome being measured, and that they could not comment on the effectiveness of each individual component in each setting. This taxonomy is a useful framework synthesising a large body of work, but of the 102 systematic reviews of self-management interventions the most common long-term conditions were asthma and diabetes [9]. Only one review of the 102 included focused on Parkinson’s, and this was a synthesis of two randomised-controlled trials (RCTs) on occupational therapy [10]. Therefore, for self-management interventions in people with Parkinson’s, which involves managing non-motor and motor symptoms, this taxonomy may not be appropriate, especially since the more recent publication of additional data on self-management in Parkinson’s [11–14].

The Medical Research Council’s guidelines on developing complex interventions emphasizes the importance of identifying key active components to ensure the best evidence-based practice in order for them to be scaled up successfully and cost-effectively [15]. Qualitative data can highlight which components of the interventions are, from the participants’ views, beneficial or unhelpful whereas quantitative data from RCTs can identify which interventions have been rigorously tested and were effective. By synthesising the qualitative and quantitative findings, interventions can be built that are more acceptable to the target population and therefore more likely to be effective [16]. One approach to integrating the qualitative and quantitative findings is to link perspectives of people with Parkinson’s with components of interventions through matrices [17–19]. A recent synthesis of qualitative and quantitative systematic reviews in palliative care using this method revealed complex multicomponent interventions were often not being conducted as participants wished (e.g., being standardised and allowing for no flexibility) [20]. The varied nature of self-management interventions may in part explain why evidence synthesis of self-management interventions in Parkinson’s has been inconclusive [13].

## Aim

To identify the key required components for self-management in people with Parkinson’s by synthesising evidence from a body of primary qualitative evidence and systematic reviews, and to explore which of these key components should be incorporated into trials of self-management in Parkinson’s.

## Methods

### Design

A mixed-methods synthesis of qualitative and quantitative data. Key components extracted from three qualitative sources was conducted including 1) interviews with people with Parkinson’s, 2) interviews with HCPs and 3) a qualitative systematic review. The synthesised qualitative data was then mapped onto the components of self-management interventions in people with Parkinson’s from a quantitative systematic review.

### Data sources

Two reviews and two qualitative studies were completed as part of the PD-Care Programme, an NIHR-funded program of work to develop an evidence-based toolkit for the self-management of Parkinson’s. Data were extracted from two recent systematic reviews on self-management in Parkinson’s: ‘*Systematic Review and Meta-Analysis of Clinical Effectiveness of Self-Management Interventions in Parkinson’s’* and ‘*Self-Management Components as Experienced by People with Parkinson’s Disease and Their Carers: A Systematic Review and Synthesis of the Qualitative Literature’* [12, 13]. For details of the methods of the systematic reviews, please see original references. Due to no UK study identified in the qualitative systematic review, further primary evidence was collected by the study team to build on the review through interviews with people with Parkinson’s and with healthcare professionals (HCP) in two qualitative studies [11, 14]. As we wanted an in-depth exploration of participants’ experiences of self-management, we focused on qualitative analysis.

#### Systematic review on effectiveness of self-management intervention in Parkinson’s disease [13]

The systematic review approach followed Preferred Reporting Items for Systematic Reviews and Meta-analyses (PRISMA) guidelines, and The Template for Intervention Description and Replication (TiDieR) was used to extract key features of the intervention [21, 22]. Eighteen randomised controlled trials (RCT) were included in the review. The aims of these trials were to evaluate self-management interventions on well-being, quality of life and function for people with Parkinson’s. All studies were conducted in high income countries and included a total of 1,645 people with Parkinson’s. The interventions varied in intensity and duration with all but one offering multiple sessions. Half of the studies assessed outcomes immediately following the intervention and the other half included a delayed follow-up. The majority of studies used primary outcome measures related to function, well-being or quality of life.

The risk of bias in the trials was high, primarily due to a lack of blinding and small sample sizes. Risk of bias is often moderate to high in these types of studies due to difficulty blinding the participants to their group. Due to heterogeneity of the trials, only a small meta-analysis on four studies was conducted, finding no significant impact on outcome, and the remaining data were narratively synthesised. The narrative synthesis revealed mixed findings: of the 18 studies, eight found a significant difference (*p* < 0.05) on primary outcomes compared to the control group at follow-up and ten did not find a significant difference. A range of components from the 18 studies were identified including information about resources; training or rehearsing psychological strategies; social support; and lifestyle advice and support.

#### Systematic review on qualitative evidence [12]

In the qualitative systematic review, six relevant qualitative studies were identified and synthesised using thematic synthesis methodology based on the guidelines by Thomas and Harden [23]. All studies were conducted in high income countries and included 104 people with Parkinson’s and 43 carers. The aims of these papers were to explore with people with Parkinson’s and their carers’ the barriers and facilitators to self-managing their Parkinson’s either specifically for an intervention or generally. The quality of the qualitative papers was judged as reasonable to good. Seven themes identified in the review included medication management, physical exercise, self-monitoring techniques, psychological strategies, maintaining independence, encouraging social engagement, and providing knowledge and information.

#### Primary qualitative evidence

Semi-structured interviews and focus groups were conducted with 42 HCPs and 21 people with Parkinson’s across two studies [11, 14]. The aim of these interviews and focus groups with people with Parkinson’s and HCPs was to explore how people with Parkinson’s are currently self-managing, the barriers and facilitators to this, and perspectives on what is needed to facilitate self-management. Reflexive thematic analysis guided by Braun and Clark’s approach [24, 25] was used to identify, analyse, and report themes. Key themes identified from these studies were empowerment of patients through holistic care and being person-centred; maximising motivation and capability for patients; including carers in self-management; management of physical symptoms; and management of the emotional impact of Parkinson’s. Please see primary papers for further details.

### Procedure

Our mixed synthesis method was informed by the Cochrane Qualitative and Implementation Methods Group’s guidance on integrating qualitative and quantitative review evidence [26] and the methods outlined in a previous synthesis of systematic reviews [20]. Specifically, to aid exploration of trial results, we selected the development of matrix table as the appropriate tool. We undertook this process in two stages.

#### Stage 1: Synthesis of key self-management components in Parkinson’s

To identify self-management components important to people with Parkinson’s and HCPs who work with them, three data sources were searched and synthesised. Firstly, the components of self-management were identified from the key themes from the systematic review of qualitative data and from the primary qualitative studies with people with Parkinson’s and HCPs [11, 12, 14]. The components were identified by the first author (MA) by searching themes and sub-themes for anything related to self-management. Secondly, the list of suggested self-management components were then confirmed in discussion with the whole study team (KW, AS, ND, DN, JR). Thirdly, components were mapped to each data source to highlight whether the component was reported in the systematic review, the primary studies or both. The components are not in a specific order nor weighted.

#### Stage 2: Mapping the qualitative and quantitative evidence in Parkinson’s

To identify whether self-management RCTs utilised the components identified in stage 1, qualitative data was mapped to the RCTs identified in a recent systematic review [27]. Firstly, intervention components from the RCTs were identified through the primary manuscript, protocol or manual if available by MA. Secondly, a matrix table was created mapping the Parkinson’s specific components identified in stage 1 to the self-management RCTs by MA and confirmed by the full study team. If the component was present in the intervention it was indicated in green. We also indicated studies that did or did not find a significant difference based on outcomes of well-being, quality of life and function, as reported as the primary outcomes in the papers and the risk of bias for each paper.

## Results

### Stage 1: Synthesis of key self-management components in Parkinson’s

Data extraction of the key themes from the three qualitative data sources (interviews from people with Parkinson’s, HCPs and a qualitative systematic review) identified 15 potential self-management components (See Table 1). As the data sources aimed to explore self-management, it was straightforward to identify potential components. The only component identified across all three sources was access to the right information and advice on Parkinson’s. Other components commonly raised were support with physical exercise, psychological strategies, increasing motivation, and social and peer support. HCPs and people with Parkinson’s appeared to focus on different aspects of self-management, with HCPs focussing on the practicality of self-management (e.g., increased opportunity and adapting to the social context) whereas people with Parkinson’s focussing more on ensuring they had the right support. These 15 components included elements needed to start self-managing (e.g., information, skill acquirement) and components needed to maintain self-managing (e.g., self-monitoring, increasing motivation).

**Table 1:**
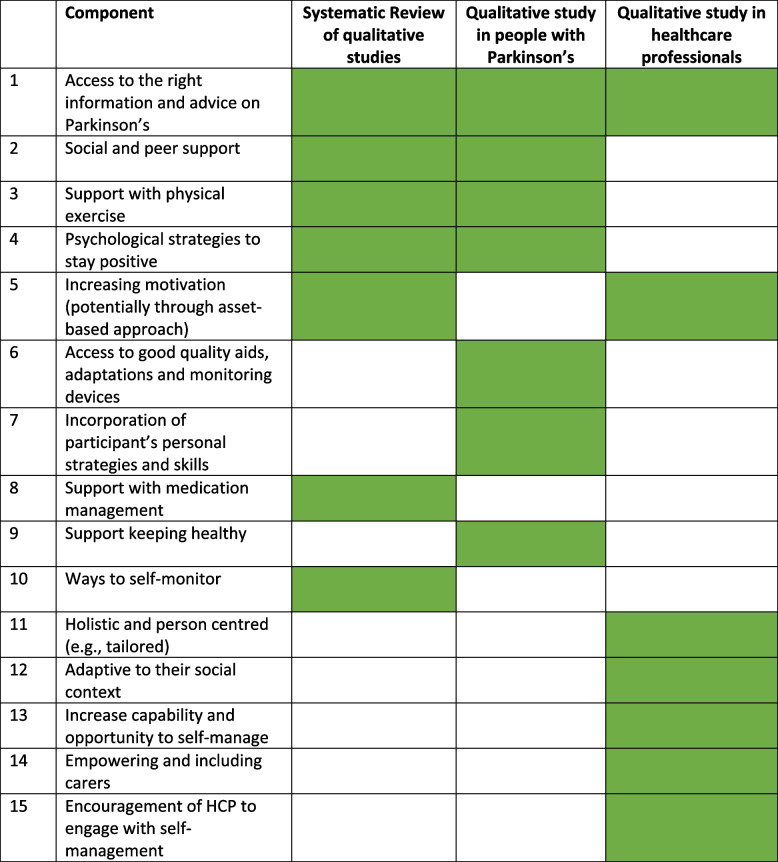
Key identified self-management components from a synthesis of evidence from people with Parkinson’s and health care professionals

Shaded colour green indicates the component was identified in the source

### Stage 2: Mapping the qualitative and quantitative evidence in Parkinson’s

The 15 components identified as important to people with Parkinson’s, carers and HCPs were mapped against findings from the results of the self-management trials in Parkinson’s in a matrix (see Table 2). This revealed that no intervention in RCTs has incorporated all the components and one component, ‘*encouraging HCPs to engage in the self-management’* which had been identified in interviews with HCP, was not part of any reported intervention trial. The other components less frequently incorporated included involving carers, increasing motivation, capability and opportunity to engage in health preventative behaviours, holistic and person-centred interventions and tailoring to the participant’s social context. Most trials without significant beneficial findings only incorporated exercise or psychological strategies along with one other component, and all these negative trials missed a number of key components (e.g., information about Parkinson’s, self-monitoring, and tailored holistic and person-centred care). Whilst none of the trials with significant beneficial effects incorporated all the components, they included a higher number of components within their interventions, with a range of 4 to 10 components compared to 1 to 3 in non-significant trials. No study that included more than 3 components had non-significant results.

**Table 2:**
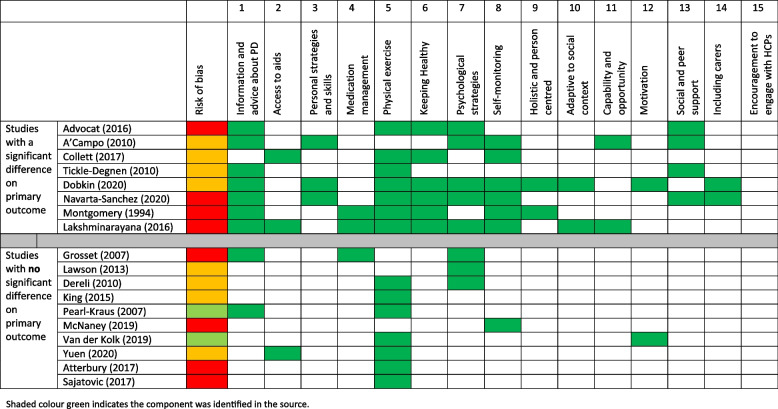
Matrix of 15 key components identified from the qualitative synthesis and their inclusion in 18 RCTs of self-management interventions in Parkinson’s (included components are indicated in green) [33–50]

## Discussion

This is the first structured synthesis of key components for self-management interventions in Parkinson’s. A set of 15 components were identified as important from the qualitative evidence and included access to information & resources, support to self-monitor the condition, psychological strategies, and social support, confirming their importance across all long-term conditions including Parkinson’s; these components are in line with the self-management taxonomy [8]. Additional components not identified in the taxonomy, included access to aids and monitoring devices, incorporation of people’s own strategies and skills, medication management, support with keeping healthy and with physical exercise, being adaptable to their lives, being holistic and person centred, techniques to increase capability and opportunity, increasing motivation, including carers, and encouraging HCPs to engage with self-management. Our matrix synthesis suggested that multi-domain interventions incorporating at least four self-management components may be more effective than more limited, focused interventions. The 15 components reflect components needed to start self-managing (e.g., information, skill acquirement) and components needed to maintain self-managing (e.g., self-motoring, increasing motivation) and should be the focus of future Parkinson’s self-management intervention or program development.

Comparing the qualitative evidence from people with Parkinson’s and HCPs with self-management trials in this population showed the components considered important by participants were more commonly present in trials that found a significant improvement on the primary outcome. We note different components were considered important by people with Parkinson’s and HCPs likely due to their differing perspectives on the disease. Caution must be made when comparing the qualitative evidence with the trial evidence as nearly all the studies had small sample sizes and potentially lacked power to detect significant outcome changes. Additionally, we do not know the quality, depth and intensity of the components provided within the trials and no study reported fidelity to the intervention. It is therefore possible that the lack of significant findings across some trials may be due to trial methodology or lack of fidelity as opposed to the intervention itself. Notwithstanding this concern, the evidence shows that the interventions that significantly improved the outcome incorporated more of the 15 components identified by people affected by Parkinson’s in qualitative interviews. Of note, many trials with non-significant findings included exercise as a component, suggesting that this alone without concomitant support to self-manage is not sufficient to improve quality of life in people with Parkinson’s.

Our findings are in line with the synthesis of evidence of self-management components for all long-term conditions with key components identified as information, psychological strategies, support adhering to treatments/medications, and practical and social support [9]. However, this synthesis identified additional components which were least incorporated in interventions of self-management trials in Parkinson’s. These included specific encouragement of HCPs to engage with self-management, involvement of carers, development of holistic and person-centred interventions, and tailoring to the social context. This finding is in line with previous criticisms of self-management interventions that they neglect involvement of the HCP, the social context, individual circumstances, and focus on the person with the condition only [46].

Developing problem-solving skills and risk-reduction behaviour have been identified as key components of self-management in Parkinson’s, as also found in the diabetes literature [47]. However, components frequently underutilised in RCTs in Parkinson’s are those that focus on the participant’s skills (e.g., asset-based approach), support to managing medication, and increasing participants capability and opportunity, and improving motivation. These are important components that are also recognised in behaviour change models; the COM-B model proposes that to change any behaviour (B) people need the physical and social capability to engage in a task (C), the opportunity to do so (O), and to want to engage in that behaviour over other competing behaviours (M) [48]. The Medical Research Guidelines advocate that interventions aiming to enable behaviour change draw on such theories in order to enhance the changes of the intervention being effective and to allow for thorough evaluation of individual components [49]. Whilst the taxonomy of self-management does not draw upon behaviour change aspects in their set of components [8], our qualitative synthesis has identified such key behaviour change components as important components of interventions for self-management of Parkinson’s.

### Strengths and limitations

The qualitative evidence included in this synthesis combined both real-life perspectives of people self-managing who have not been included in self-management trials with the views of participants from self-management trials from process evaluations. Including both sources of information allow for direct evaluation of people who have tested self-management intervention as well as ‘real world’ data. Integrating findings from both quantitative and qualitative systematic reviews is a relatively new area and there is no ‘gold standard’ method. However, we used existing advocated approaches to ensure the transparency and replicability of our methods. Our findings are limited by the quality of the primary evidence from the quantitative review and under reporting of components of interventions within studies. Additionally, a range of different interventions were included in the quantitative systematic review; however, many studies used few components from the self-management taxonomy or from the standard definition of medical, emotional and behavioural aspects [6].

### Further research and clinical implications

Future research should consider testing the appropriateness of the 15 self-management components in Parkinson’s across different contexts such as different locations and different stages of Parkinson’s. Additionally, it would be important to identify if any of the 15 components have more of an impact on outcomes than the others. The importance of behaviour change aspects as identified by the participants highlights the need for interventions that target all three aspects of self-management: physical, emotional, and behavioural. Co-designing interventions with people affected by the condition is increasingly used to produce an intervention that meets the needs and is acceptable to the end users, and therefore more likely to be adopted and sustained [50]. Our findings suggest that such interventions should be multi-domain and incorporate multiple components that support both initiating and maintaining self-management.

## Conclusion

This synthesis of data has identified 15 key components that support initiation or maintenance of self-management for people with Parkinson’s that can be incorporated into interventions or programs. These should include behaviour change techniques and incorporate context. Whilst none of the trials with significant effects integrated all the components, those with significant results included a higher number of components within their interventions (four or more) highlighting the importance of incorporating a range of components to help people self-manage Parkinson’s. Further research should focus on co-designing interventions for people with Parkinson’s incorporating these components.

## Data Availability

Data used in this study is published in primary studies in the links below. HCP data: https://bmcgeriatr.biomedcentral.com/articles/10.1186/s12877-021–02678-w People with Parkinson’s data: https://www.ncbi.nlm.nih.gov/pmc/articles/PMC9462566/ Systematic review of quantitative data: https://bmcgeriatr.biomedcentral.com/articles/10.1186/s12877-021–02656-2 Systematic review of qualitative data: https://pubmed.ncbi.nlm.nih.gov/33489082/
